# Becoming an Autologous Stem Cell Transplant Patient

**DOI:** 10.1093/geroni/igaa057.382

**Published:** 2020-12-16

**Authors:** Sean Halpin, Michael Konomos, Ivey Jowers

**Affiliations:** 1 University of Georgia, Decatur, Georgia, United States; 2 Emory University, Atlanta, Georgia, United States; 3 Winship Cancer Institute, Atlanta, Georgia, United States

## Abstract

In the current study, we sought to examine how older patients incorporate the identity of a patient receiving autologous stem cell transplant (ASCT) for multiple myeloma (MM) into their daily lives. In this ethnographic study using interpretative phenomenological analysis, we observed pre-transplant education visits with 30 MM patients, followed by semi-structured interviews in their hospital rooms during transplant. The experience of receiving ASCT for MM required effort by patients to not only maintain their past identity but also establish a new patient identity. Reconciling these two identities required deliberate and emotionally draining effort from the patient. Results were organized into two overarching themes of social relationships and aesthetics with subthemes for each. Patients experienced challenges reconceptualizing their social support network to meet their changing needs; often with a spouse or child taking on a caregiving role. In regard to aesthetics, patients contended with the physical reminders of their new diseased identity, adopting various aesthetic strategies to either embrace or conceal bodily changes. Understanding methods MM patients who are receiving ASCT use to negotiate normalcy during treatment may be helpful for developing interventions for alleviating distress during this difficult time.

